# Supplementary material for: Heterostructures formed through abraded van der Waals materials
Source: Nat Commun. 2020 Jun 16;11:3047. doi: 10.1038/s41467-020-16717-4 (PMC7297739; doi:10.1038/s41467-020-16717-4)
Supplement: Supplementary file 1 — Supplementary Information [file 41467_2020_16717_MOESM1_ESM.pdf]

Supplementary Information

Heterostructures formed through abraded van der Waals materials

Darren Nutting et al.

Supplementary Note 1: Additional scanning transmission electron microscopy

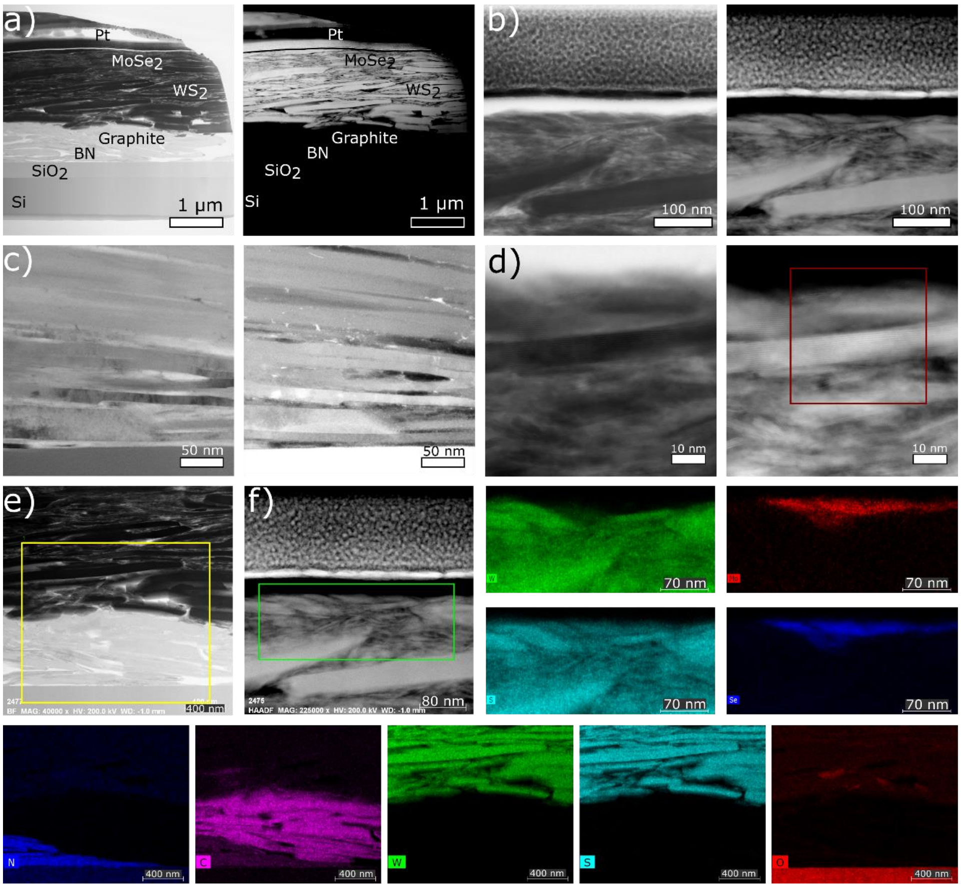

Supplementary Figure 1. Scanning transmission electron microscopy (STEM) structural characterisation of cross sectional lamella produced from vdW heterostructures synthesised through mechanical abrasion (fabrication route 1). a-d. Bright field (BF), left, and annular dark field (ADF), right, STEM images. The different layers are indicated in **a.**, identified from elemental mapping (Fig. 1b). Different contrast arises from differences in scattering due to variations in nanocrystal orientation, atomic number and density. **e.** Cross-sectional HAADF image of the bottom three layers of the heterostructure with accompanying elemental maps shown underneath. The yellow square highlights the area from which these maps were taken. A Pt strap is visible at the top of (**a**,**b**, and **f**), and was used to prevent specimen damage during focussed ion beam milling.

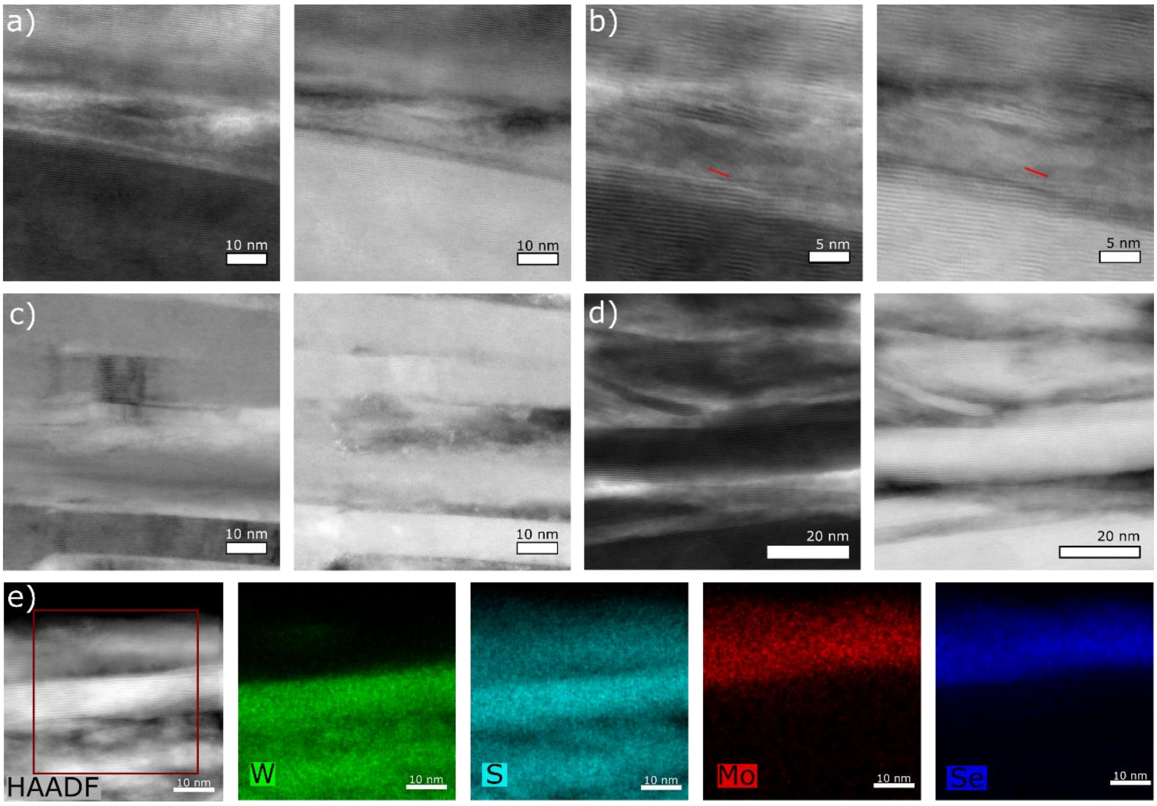

Supplementary Figure 2. High resolution STEM characterisation of vdW heterostructures synthesised through mechanical abrasion. a. b. c. d. Cross-sectional bright field (BF), left, and annular dark field (ADF), right, images, displaying the $MoSe_2$-WS_2 heterostructure interface, graphite layer **c,** and hBN layer **d**, respectively. Visible lattice fringes demonstrate the expected lattice spacing for the different vdW structures and the local alignment of neighbouring nanocrystals produced by the mechanical abrasion processing. **e**. ADF image and STEM-EDS elemental maps of the $MoSe_2$-WS_2 heterostructure interface region highlighted by the red square.

Supplementary Note 2: Size analysis of flakes deposited via mechanical abrasion.

As can be seen in Supplementary Figure 3, the dimensions of mechanically abraded crystallites are similar regardless of the material used, with thickness of ~ 10-40 nm and lateral sizes of ~ 10-400 nm, which is significantly thicker and smaller than sheets produced through liquid-phase exfoliation[1]. The thicker individual crystallite size is the likely leading factor for the improved performance compared to liquid phase deposited films.

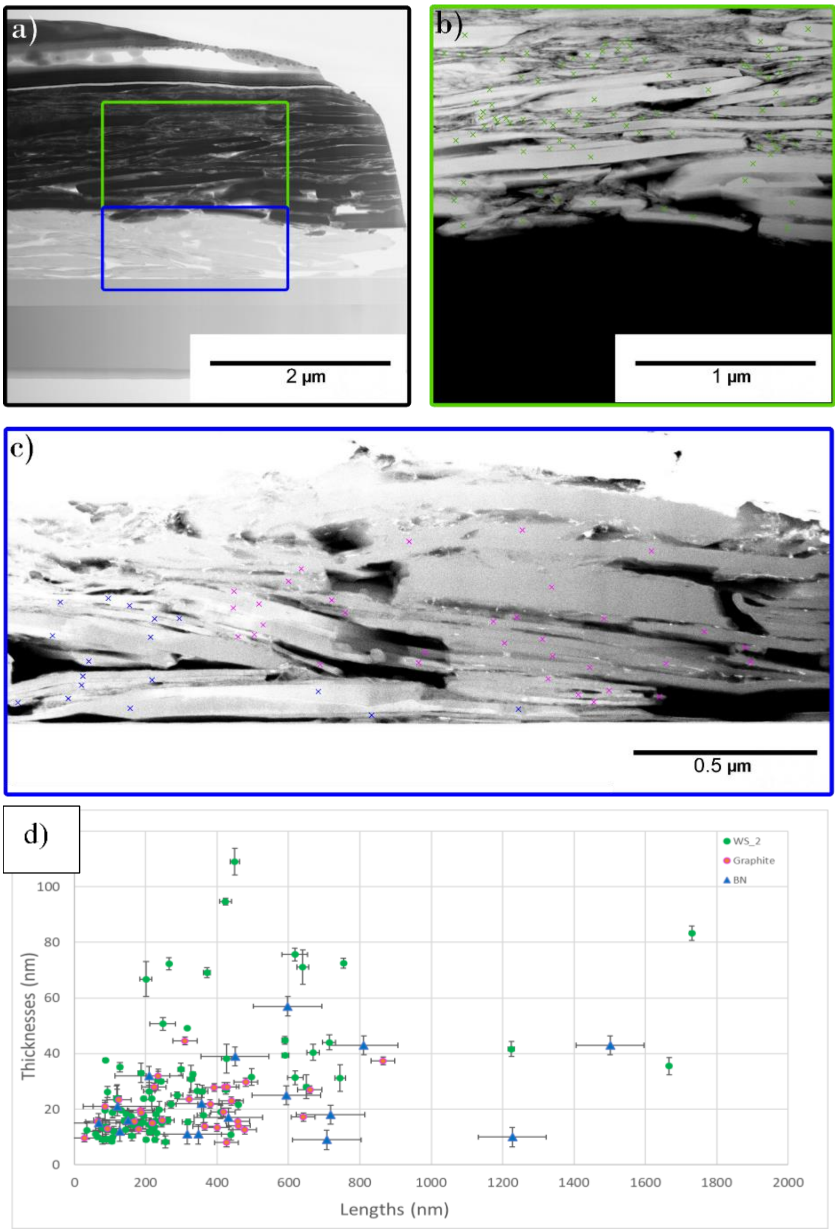

Supplementary Figure 3. Size analysis of flakes deposited using mechanical abrasion. a-c. Cross-sectional scanning transmission electron microscopy (STEM) imaging of the deposited vdW heterostructure. **a.** Bright field STEM image of the full film thickness, with **b,c**. annular dark field STEM images for the rectangular regions indicated in **a**. Green crosses indicate separate WS_2 flakes, while pink and blue crosses highlight graphite and boron nitride flakes, respectively. **d.** Corresponding thickness and length distributions for more than deposited flakes. This analysis is performed with a spatial resolution of ~10nm in order to sample a sufficiently large number of flakes but higher magnification imaging reveals that many thinner flakes are also present in the deposited material. Error bars in **d** correspond to the statistical variation observed within repeated manual measurements of each flake.

Supplementary Note 3: Example device fabrication

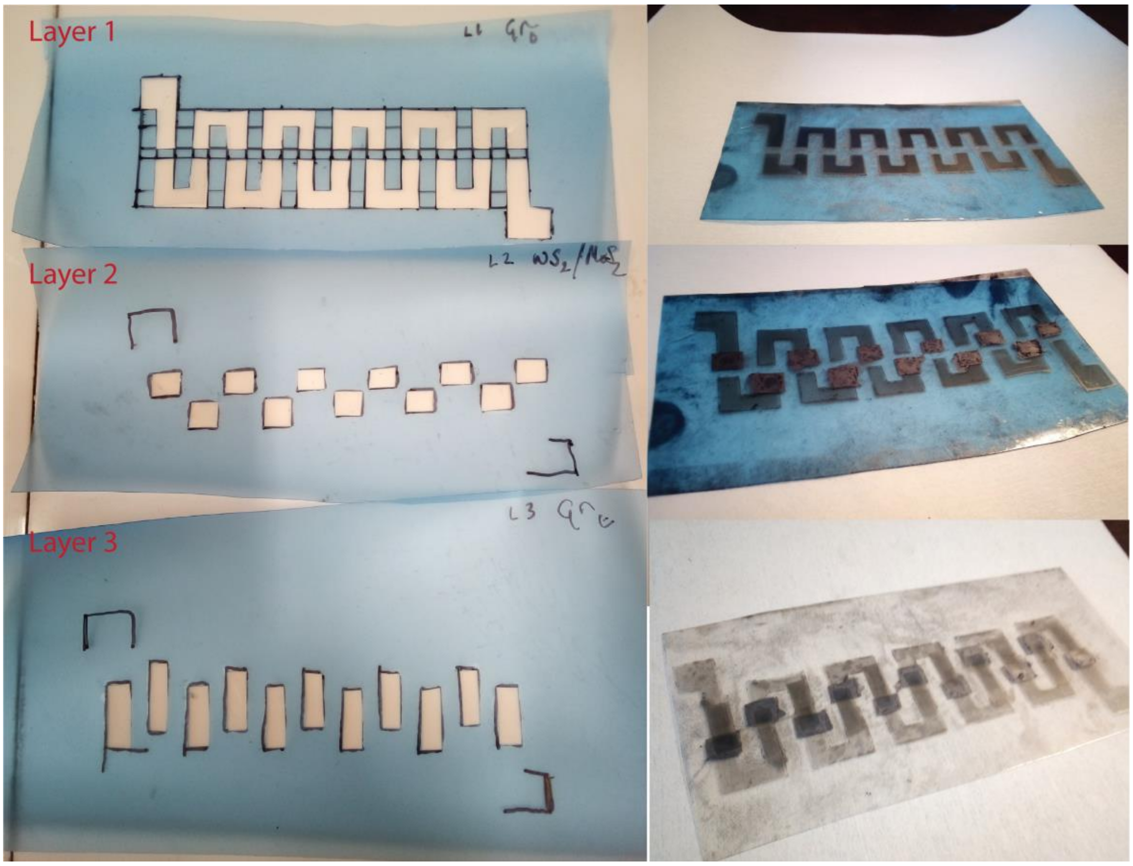

Supplementary Figure 4. Example of the multilayer heterostructure production via a tape template.

The general fabrication process for the multilayer heterostructures is shown in Supplementary Figure 4. Device fabrication is carried out by cutting templates into the tape and abrading the 2D crystals onto a PET substrate in selected locations. Removal of the tape mask leaves the abraded films in selected locations only. The process can then be repeated to build up vertical heterostructures of arbitrary device dimensions. It was found that when creating vertical heterostructures, which are many layers thick and also have a top and bottom graphite layer, it was advantageous to follow fabrication route 2 (highlighted in Fig. 1a of the main text) to prevent short circuit between the two electrode layers.

Supplementary Note 4: Transmission spectra of abraded van der Waals materials

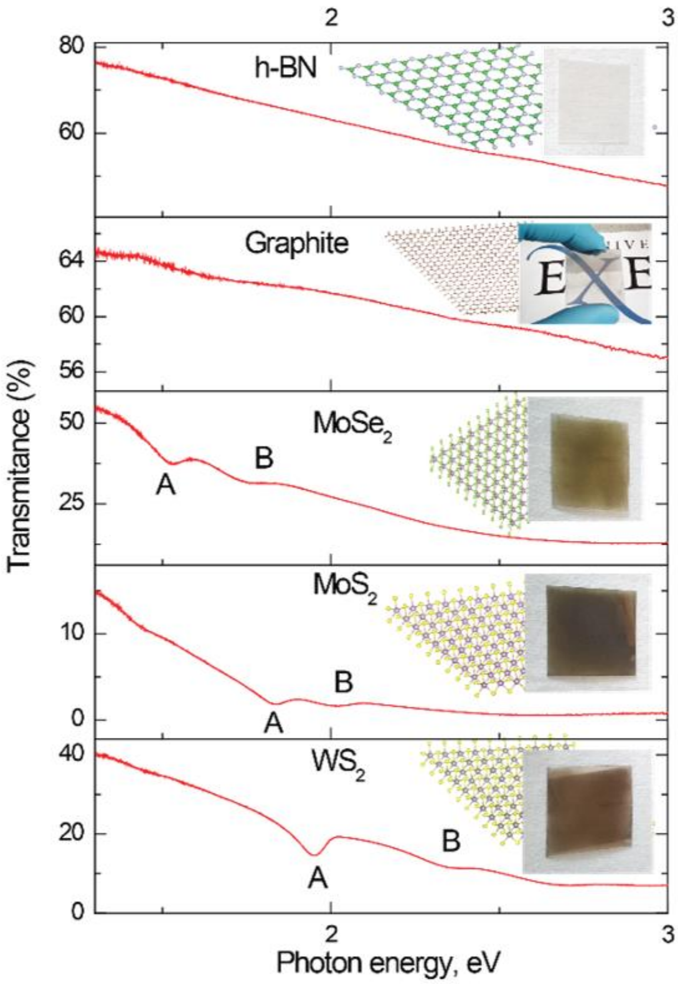

Supplementary Figure 5. Optical transmission spectra for vdW material coatings on PET substrates.

Supplementary Figure 5 shows the optical transmission spectra of several different abraded vdW material coatings on a 0.5 mm thick PET substrate. The resultant films still display transparency which can be tuned to higher levels by back peeling the films with an adhesive tape. For the transition metal dichalcogenide films, strong absorption associated with the A and B excitons are still observed, indicating that the films still display similar optical properties comparable to the bulk pristine materials.[2, 3]

Supplementary Note 5: Additional optoelectronic devices and measurements

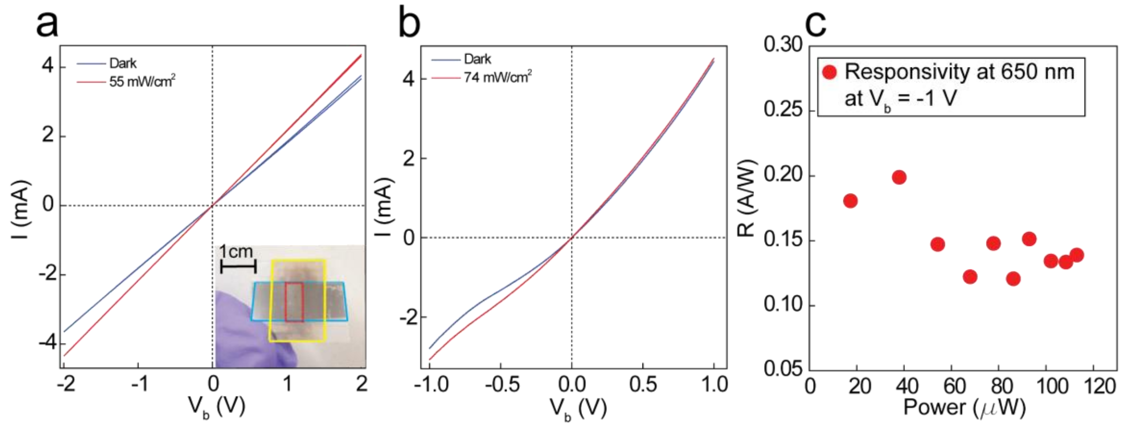

Supplementary Figure 6. Additional data for the devices described in Fig. 2. a. I-V_b characteristics for the WS_2 planar photodetector with and without white light excitation. Inset: image of device with blue region: graphite, red region: strip of graphite thinned with tape back peeling, yellow region: directly abraded optically active TMDC top layer. **b.** Zoom in of the I-V_b curves for the vertical Au-WS_2-(CVD graphene) device described in Fig. 2B with and without optical excitation. **c.** Photoresponsivity for the same device as shown in Fig, 2b measured using a 10 nm bandpass filter centred at 650 nm for increasing excitation power.

Supplementary Figure 6, shows additional electron transport and photoresponsivity data for the devices described in Fig. 2 of the main text. The inset shows one of our devices, where we used an adhesive tape to back peel the graphite in order to increase its transparency before abrading the TMDC top layer onto the graphite. This allows us to optically excite the device through the PET side, increasing the amount of light incident on the graphite-TMDC interface.

Supplementary Figure 7a shows the response time for a simple planar photodetector based on WS_2 and biased to V_b=50 mV. The I-V_b curves for this device are also presented in Supplementary Figure 8. We also produced a device with an amorphous carbon (a-C) top electrode deposited through candle flame deposition[4], however we found that this device

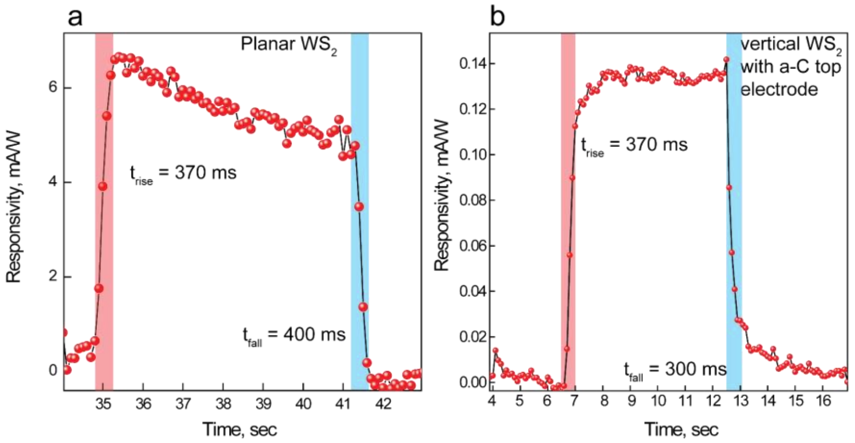

Supplementary Figure 7. Response times of different photodetectors. a. Planar WS_2 junction at V_{sd}=50mV. **b.** Vertical WS_2 junction with a-C top electrode measured at V_{sd}=1 V.

displayed a poor responsivity due to the low transparency a-C top electrode, Supplementary Figure 7a.

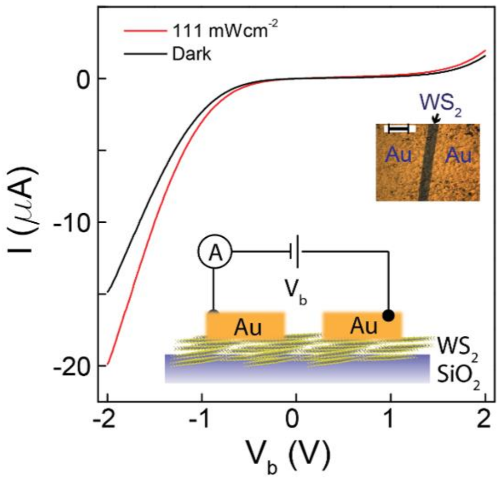

Supplementary Figure 8. Mechanically abraded films for photodetection applications. I-V_b for a planer geometry WS_2 channel without (black curve) and with (red curve) 111 mW/cm^2 incoherent optical excitation (length = 25 mm, Width= 5 mm) (scale bar = 50 mm).

Photodetectors were also produced using p and n-type silicon electrodes. To produce those devices, we etched away a 1 cm^2 region of SiO_2 in a sodium bifluoride etch solution[5] leaving a clean Si surface. The WS_2 was then abraded over the n/p type silicon until no pinholes could be observed optically under a 50x objective. CVD graphene was then transferred over the WS_2 and baked for 30 min at 100 °C so that the PMMA adheres well to the WS_2. For electron transport measurements the PMMA was left on, which may have helped bridge any pinholes in the WS_2 film, preventing short circuit. The resistance of the CVD graphene is shown in the inset of Supplementary Figure 9a and corresponds to R= 670 W. Typical I-V_b curves obtained in the dark and under white light illumination are shown in Supplementary Figure 9a with the short circuit current and open circuit voltage shown in Supplementary Figure 9b. Slightly improved performance was observed for p-type silicon (expected as the WS_2 is n-type,

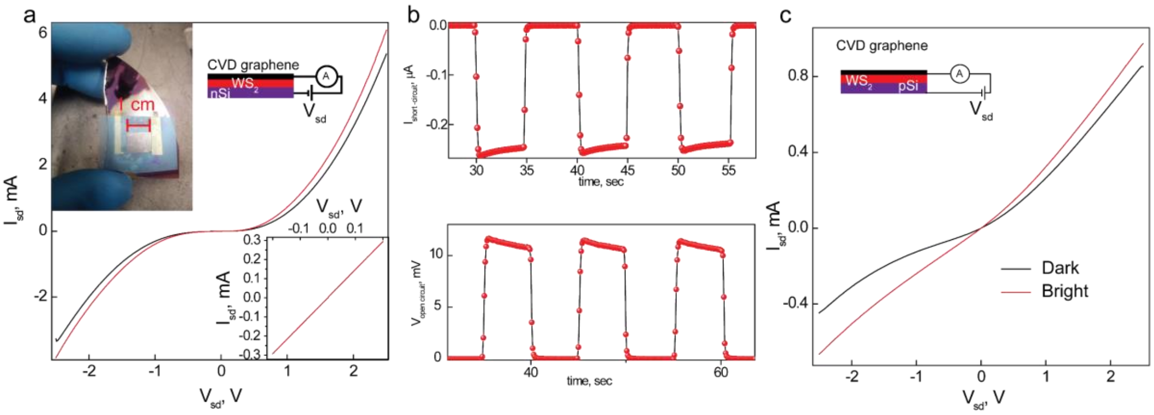

Supplementary Figure 9. Photovoltaics with silicon. a. Vertical I-V curves for a n-Si / WS_2 / CVD graphene device. Inset: (bottom right) I-V of the CVD graphene top electrode R=650 Ω (top left) image of the device (top right) measurement circuit. **b.** Open circuit voltage and short circuit current for the device shown in **a**. **c.** Vertical WS_2 junction but this time with p-type boron doped silicon.

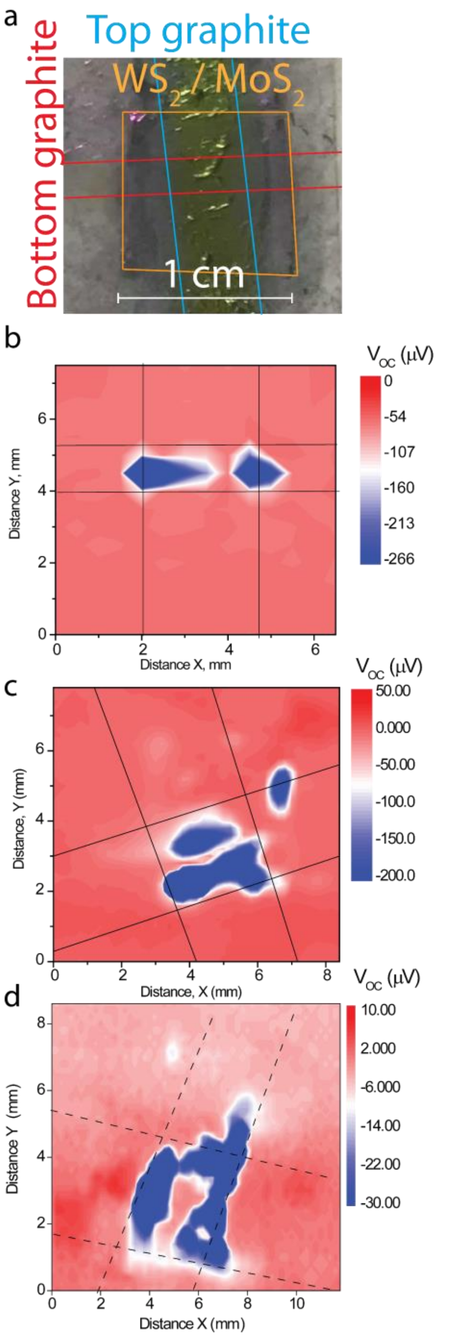

Supplementary Figure 10. Photo-voltage mapping for additional devices. a. Photograph of a typical device. **b-d.** open circuit photo-voltage cntour maps of three additional devices.

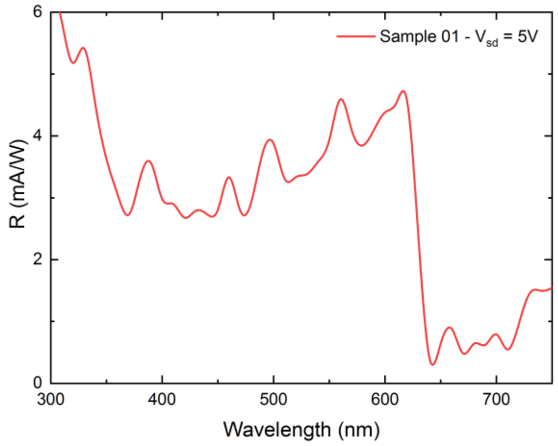

Supplementary Figure 11. Spectral dependence of the responsivity for the MoS_2 planar detector described in Fig. 2A

confirmed from previous transport measurements). However, overall these devices did not work well due to the TMDC thickness, ~0.3-1 mm. This prevents a significant amount of light reaching the depletion region. Therefore, most charge separation likely takes place at the graphite-WS_2 Schottky junction region, resulting in the weak observed signal.

Supplementary Figure 10, shows an optical photograph of a vertical photovoltaic device based purely on abraded 2D materials along with three additional photovoltage maps for graphite-WS_2-MoS_2-graphite devices created through fabrication process 2 as described in the main text of the paper. The devices all show predominant photovoltage over the overlap region between the bottom graphitic electrode, the TMDC barrier layers and the top graphitic electrode. The device shown in Fig. 2f of the main text has been annealed to 150 degrees in atmosphere for 60 min and consequently shows more uniform

photovoltage over the device area due to the better contacting quality of the top graphitic electrode.

Supplementary Figure 11, shows an additional high spectral resolution photo-responsivity data for the MoS_2 planar junction described in Fig. 2A of the main text. This was recorded in a

**Active material/Detector type/Architecture**	**Key parameters: Environment/Bias / power**	**Photo-responsivity**	**Our like-for-like responsivity**	**Abraded device enhancement factor**
**LPE-WS_2/Vertical, all printed graphite contacts (best device on PET)[5]**	Ambient/-0.5 V/2.76 mW (Laser)	36 μA/W	24 mA/W	667
**LPE-WS_2/Vertical all printed graphite contacts with 1 Ohm/sq resistance[40]**	Ambient/-0.5 V/ 334 mW/cm^2 (Scale on the figures made it hard to resolve the current change at low biases)	~10 mA/W	5 mA/W	500
**Gr-WS_2-Gr/Liquid exfoliation, mechanically exfoliated graphene and Au contacts[21] (30 nm WS_2 film was used in this study. Non-Scalable production)**	Ambient/-1 V/ 37mW (focussed laser)	0.7 mA/W	0.15 A/W (We compare to our Au-WS_2-Gr device as is has the thinnest WS_2 barrier of all devices studied and similarly has monolayer CVD graphene top electrode.)	214
**LPE-WS_2/ Planar, fully printed[32]**	Ambient/-1 V/<800 mW/cm^{-2}	0.1 μA/W	10 mA/W (compared to the WS_2 channel in fig S8 at V_b=-1V)	1.0 x 10^5
**LPE-WS_2/Planar, fully printed[34]**	Ambient/-1V/12.5 mW	0.56 μA/W	10 mA/W (compared to the WS_2 channel in fig S8 at V_b=-1V)	1.7 x10^4

Supplementary Table 1. Comparison of photoresponsivity of abraded devices to those reported in literature fabrication through different approaches.

separate measurement system and yields a similar responsivity as presented in the main text. The larger noise seen here is due to a much lower power density of the lamp at each wavelength which was in the mW/cm^2 region. Supplementary Table 1, compares responsivities of different vdW material photodetector technologies. Our devices typically yield a significant enhancement compared to scalable liquid phase exfoliated devices. They also offer an enhancement of ~10^2 times when compared to non scalable liquid phase devices with ultra-thin

(~ 30 nm thick) WS_2 barriers[6]. However, our values of responsivity are still a factor 10-100 worse than those values observed in similar devices produced through mechanical exfoliation and transfer of single crystals[7]. We aim to enhance the performance through optimisation of post fabrication annealing of the devices as this could improve the interfacial quality between the different nanocrystal layers.

Supplementary Note 6. Raman spectroscopy and atomic force microscopy

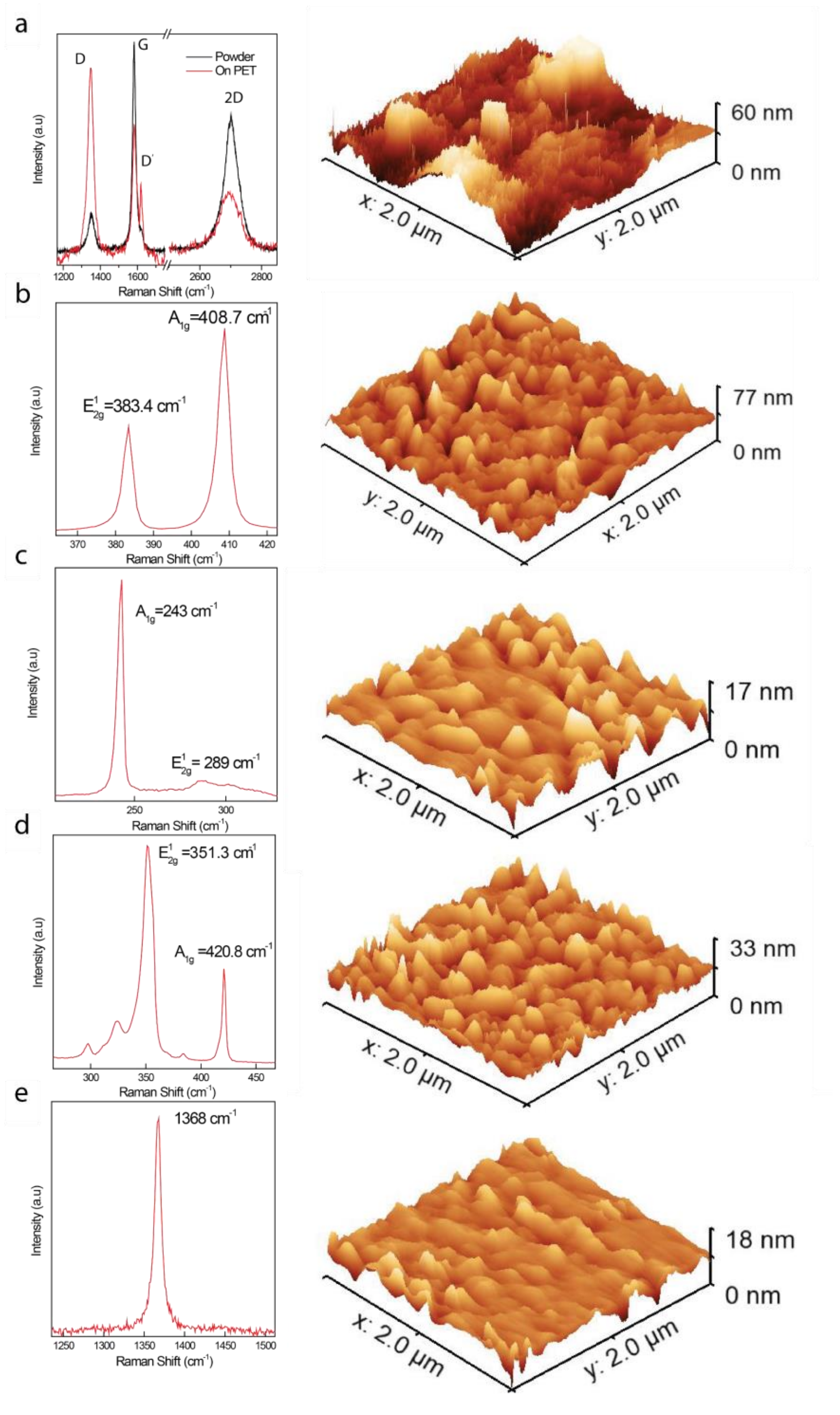

Supplementary Figure 12. Raman spectra and AFM. a-e. (left) Raman spectra and tapping mode AFM scans (right) for graphite, MoS_2, $MoSe_2$, WS_2 and hBN on respectively.

Raman spectroscopy is an important tool for the characterisation of vdW materials. Supplementary Figure 12a (left) shows the Raman spectrum of graphite powder and abraded graphitic films on a PET substrate. The normal Raman modes are observed in both cases. The 2D peak at 2711 cm^{-1}, G mode at 1581 cm^{-1} and D mode which is associated with disorder at 1350 cm^{-1}. The ratio of the intensity of the G-mode (I_G) to the D-peak (I_G) gives a measure of the disorder in our graphitic films. It should be noted that the split peak seen in the G -mode is an artefact of the removal of the PET background which has very closely positioned peaks to the graphite signal and not the D’ peak commonly seen at that energy. In our graphitic powder samples, we obtain

Material	Roughness (nm)	Error (nm)	Thickness (nm)
**MoS_2**	26.4	5	80-90
**$MoSe_2$**	4.2	2	40-70
**WS_2**	6.0	2	200-300
**hBN**	9.4	3	80-90
**Graphite**	22.7	10	20-30

Supplementary Table 2. Estimates of surface roughness of various abraded films

an increase of the I_D/I_G=0.20, with an increase of the disorder (reduction of crystallite size) for the abraded film I_D/I_G=1.43. The Raman spectra for the TMDC films and hBN are all found to be comparable to the literature values for bulk crystals (Supplementary Figure 12(b-e) (left)), see refs[8, 9, 10, 11].

Estimates of thickness and RMS roughness of each material used are shown in Supplementary table 2, and were calculated using Gwyddion analysis software[12] The typical thicknesses of the abraded films were measured using an Alpha-Step D-100 Stylus Profiler. We note that the thickness of films vary significantly over the entire film, leading to a large uncertainty in the film thickness.

Supplementary Note 7. SEM images of the films

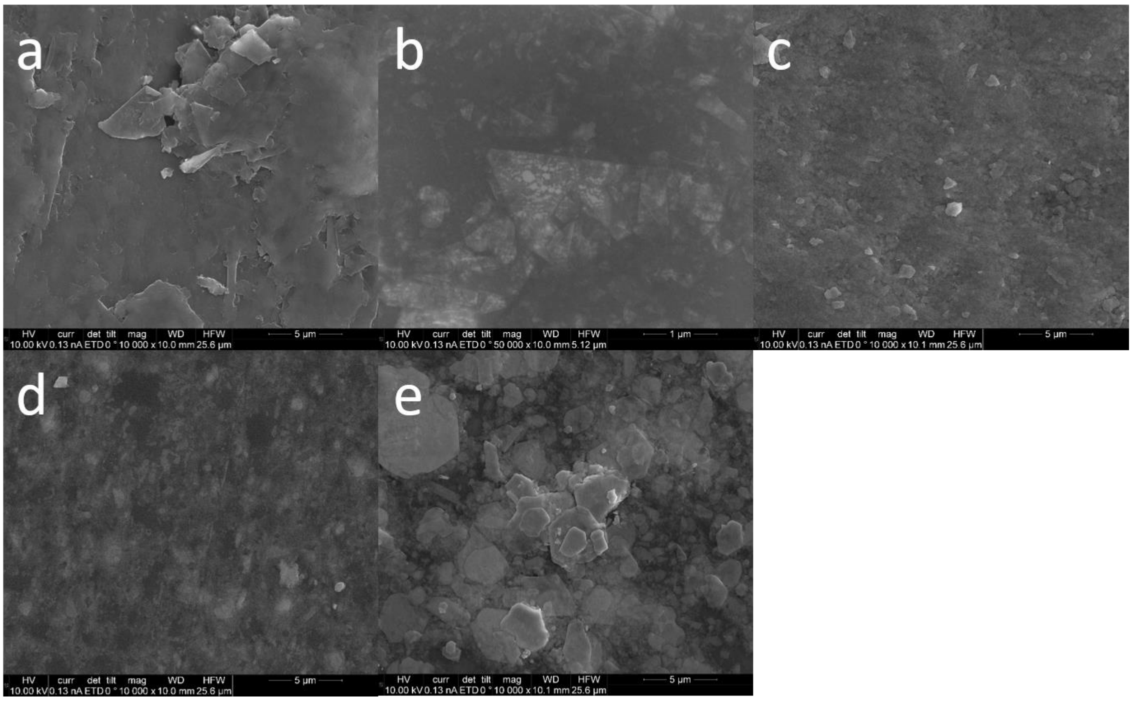

Supplementary Figure 13. SEM images. SEM images of **a**. graphite (on PET), **b.** hBN, **c.** MoS_2, **d.** $MoSe_2$ and **e.** WS_2 abraded films on a Si/SiO_2 substrate.

Supplementary Note 8: Sheet resistance, transmission spectra and comparison of in and out of pane resistivity

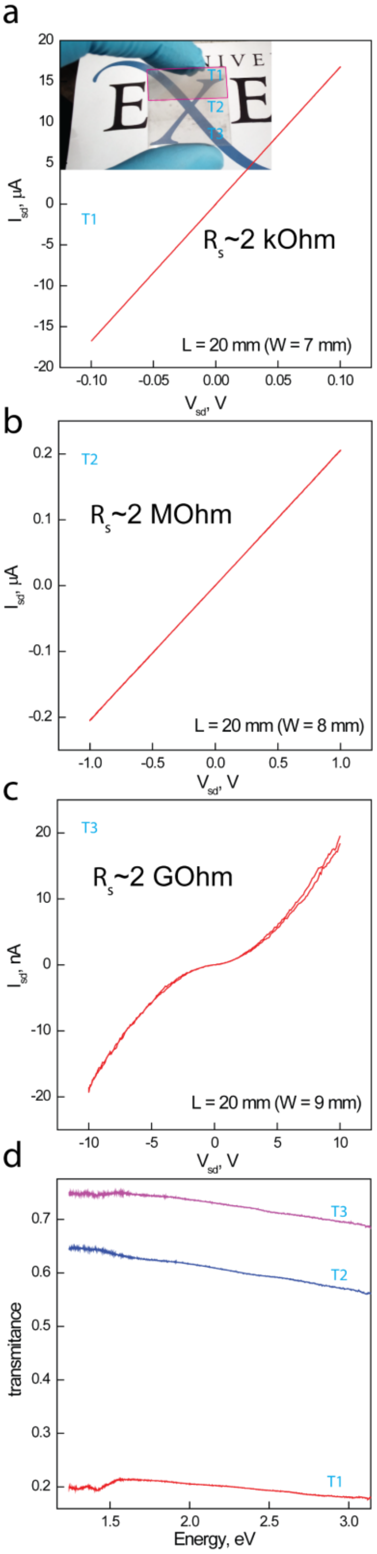

Supplementary Figure 14. Graphite resistance vs transparency. a-c. I-V curves for graphitic films of different thickness. **d.** Transparency of the films measured in **a-c**.

The I-V curves for three different transparency graphitic films are shown in Supplementary Figure 14(a-c). The transparency is increased by using specialist tape to thin the thickness of the graphitic films. We find a strong dependence of the film thickness with sheet resistance, increasing by 3-orders of magnitude for each additional peel away of the graphitic film. It is expected that the sheet resistance could be reduced further through chemical intercalation or doping methods[13]

The optical transmittance of the films measured in Supplementary Figure 14(a-c) is also presented in Supplementary Figure 14d.

Supplementary Figure 15, shows a plot of the transparency vs resistance for different abraded graphite films. We find comparable performance to transparent electrodes prepared through shear force exfoliation[14].

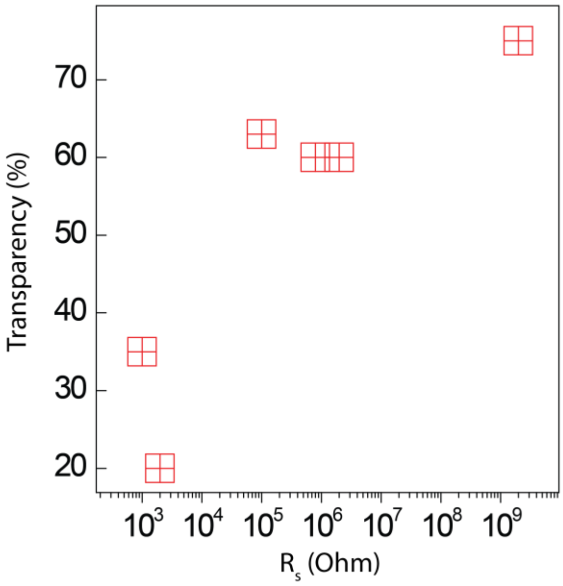

Supplementary Figure 15. Sheet resistance vs transparency for different abraded graphite films.

Supplementary Table 3 compares the resistivity of our films to exfoliated, CVD, mechanically exfoliated and liquid phase TMDC systems. We must stress that this comparison could be erroneous due to different conditions (e.g. charge carrier density, temperature, environment etc...). We have surveyed the literature and compared measurments taken at similar conditions and device type to ours. We find that the resistivity of our TMDC films are more comparable to mechanically exfoliated[15], CVD grown crystals[16] or bulk properties[17] . While our films consistently show significantly lower resistivity by a factor 10^4-10^5 when compared to liquid phase films[18, 19, 20].

Device type and reference	In-plane Resistivity at V_b = 1 V (Wm)	Comments
**Abraded WS_2 film (This work)**	17 - 50	Two-terminal measurement with Au electrodes deposited onto the WS_2 through a shadow mask. Large range due to uncertainty in the WS_2 film thickness (0.5 to 1.5 mm)
**Printed MoS_2 detector[33]**	4 x 10^6	This value is taken from the paper text and is not possible to infer the exact resistivity at V_b= 1 V. It also has printed graphene contacts opposed to evaporated Au.
**Printed WS_2 film[34]**	1 x 10^5	All printed WS_2 film. Parameters used from [36], I(2V)=2 nA, L= 1 cm, t = 1 mm, W= 50 mm
**Mechanically exfoliated few layer WS_2 transistor with Au electrodes[15]**	0.1 - 1	Rescaled sheet resistivity to 3D resistivity for doped bilayer WS_2.
**CVD grown WS_2 with graphene electrodes[16]**	5-15	Rescaled sheet resistivity to 3D resistivity for undoped WS_2.
**Bulk resistivity at Room temperature[17]**	10-100	$n \sim 10^{16}$ cm^{-3}

Supplementary Table 3. Comparison of the bulk in-plane resistivity of our films compared to films produced through other methods.

A similar comparison can also be made of the vertical devices which results in a similar trend. For instance, the vertical resistivity for multilayer mechanically exfloiated WS_2 varies from 1.25×10^7 to 12.5 Wm[21] depending on the doping level of the WS_2 crystal at a bias voltage of V_b = 0.2 V. Whereas mechanically constructed liquid phase exfoliated WS_2 vertical transistors display resistivity of 1×10^6 Wm at V_b = 0.25 V[6] with printed heterostructures also displaying very similar vertical resistivity for devices on PET and paper[22]. In this work we obtain values of vertical resistivities of $(1.5 \pm 1) \times 10^5$ Wm at V_b = 0.25 V for our WS_2/MoS_2 junctions (Fig. 2D, device 1, thickness=1 mm, W~2 mm and L~4 mm) taking into account the error in the film thickness and errors in determining the contact area. If we analyse the resistivity at higer

bias, say V_b=0.5 V to the fully printed heterostructures[22], we find that the resistivity becomes significantly lower in our devices ~40 kWm vs ~400 kWm as the current increases faster for increasing V_b. We note that this could be due to the printed graphitic contacts dominating the resistivity. However, overall our films display lower resistivity at a given applied electric field compared to liquid phase materials. However, a systematic study including Hall effect measurements are required to accurately compare materials produced from different methods.

Supplementary Note 9: Large area films on stainless steel for the hydrogen evolution reaction

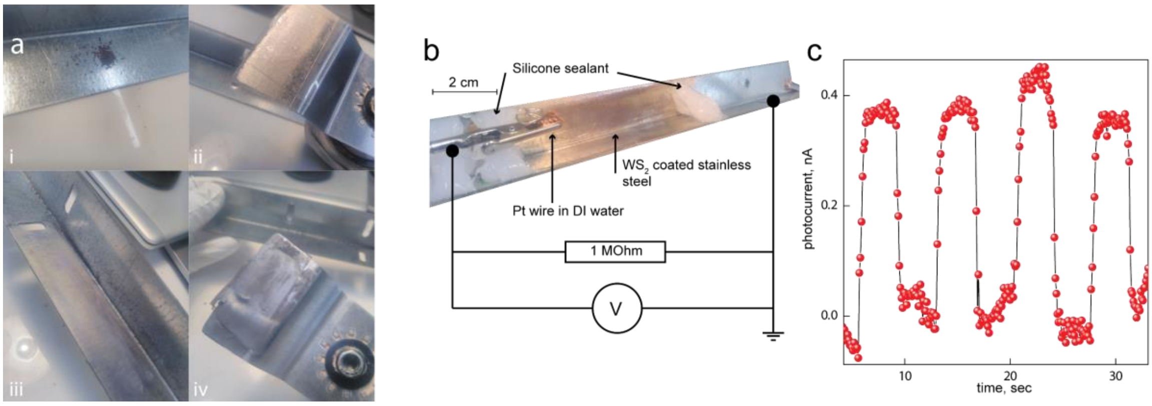

Supplementary Figure 16. HER on stainless steel. a. Images of the large-scale deposition method. **b.** Schematic of the electrical setup and device under illumination. **c.** The photocurrent measured through a 1 MOhm resistor when illuminated with a white light source of ~10 mW/cm^2.

For large area films we made with the use of a multi-tool instrument, specifically a Titan TTP448HT with oscillation speed of 15000 vibrations per min. Essentially, a PDMS pad is placed on the tool and the vdW powder is placed between the tool and the substrate. The vdW crystal pad then rapidly oscillates back and forth to deposit a thin abraded film onto the substrate of choice. Supplementary Figure 16, shows the general steps for the deposition of a WS_2 film on a piece of stainless-steel angle bar.

To demonstrate its general use for HER we sealed a region with the WS_2 film with a silicone sealant, which was subsequently filled with DI water (i.e. a makeshift gutter). The photocurrent through a 1 MOhm resistor was then recorded and is displayed in Supplementary Figure 16c for 4 illumination cycles of the incident white light illumination. We find that this method is better suited for large area deposition and could easily be up-scaled easily to the m^2. Supplementary Figure 17 displays a second catalyst coating on an Au substrate in DI water.

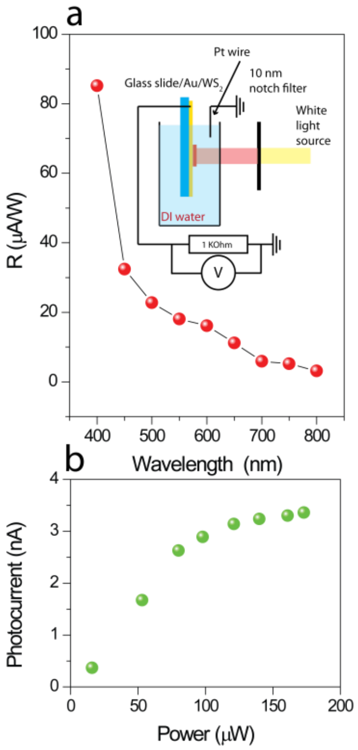

Supplementary Figure 17. WS_2 catalyst on Au substrate. a. Spectral dependence of the responsivity for a WS_2 catalyst abraded on Au in DI water. **b.** power dependence of the photocurrent at 550 nm excitation.

Supplementary Figure 17a displays the responsivity for different optical excitation energy. Here, the optical power is recorded for each notch filter and the responsivity can then be determined from measurements of the photocurrent. We find the peak responsivity is found for low powers and rapidly saturates for excitation powers in excess of 75 $mWcm^{-2}$.

Supplementary Note 10: Additional Strain measurements and bending rig setup

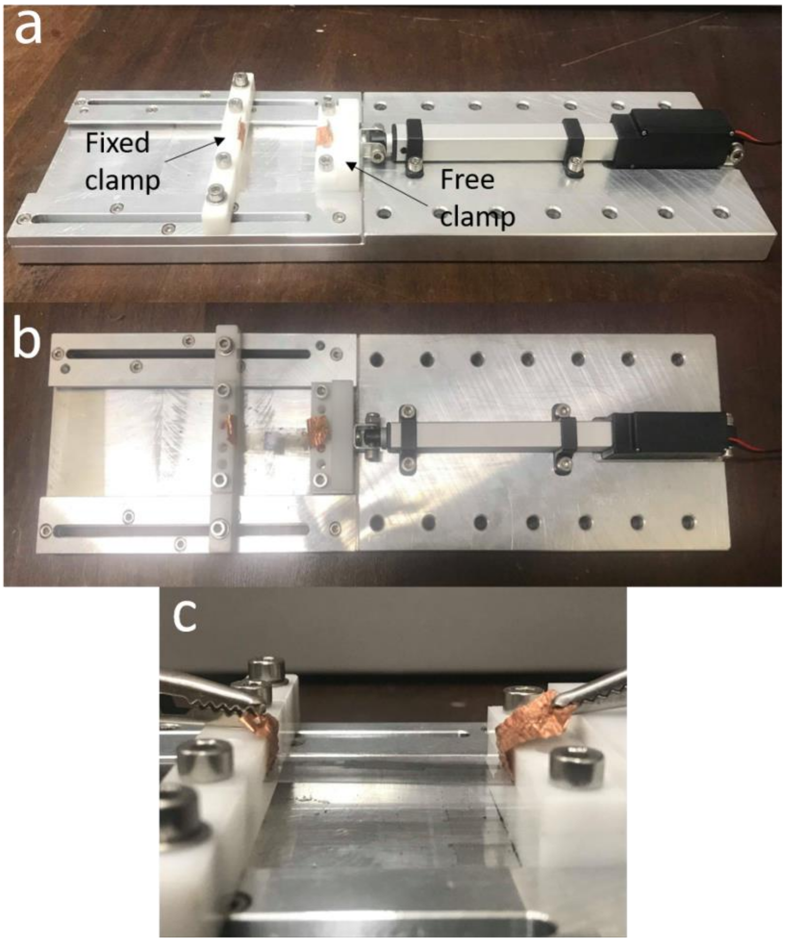

Supplementary Figure 18. Image showing the rig used to apply strain to the devices. **a**. and **b.** show the bending rig before and after loading a device. **c.** A zoom in of an unstrained and contacted device within the bending rig.

Supplementary Figure 18, shows the bending rig set up used to apply uniaxial strain. The devices are held in place by two clamps, one of which is fixed in position to the underlying plate. An Actuonix L16 Micro Linear Actuator is used to push the free clamp forwards in order to bend the sample and apply strain. Dovetail grooves are used to ensure the free clamp can only move parallel to the direction of the actuator arm. The actuator is driven by a DC voltage pulse and the distance moved by the clamp can then be used to calibrate the rig. The distance moved and therefore strain applied can then by accurately controlled by tailoring the time of the voltage pulse driven through the actuator. Conducting copper tape is used to provide electrical contact to the devices for two terminal resistance measurements.

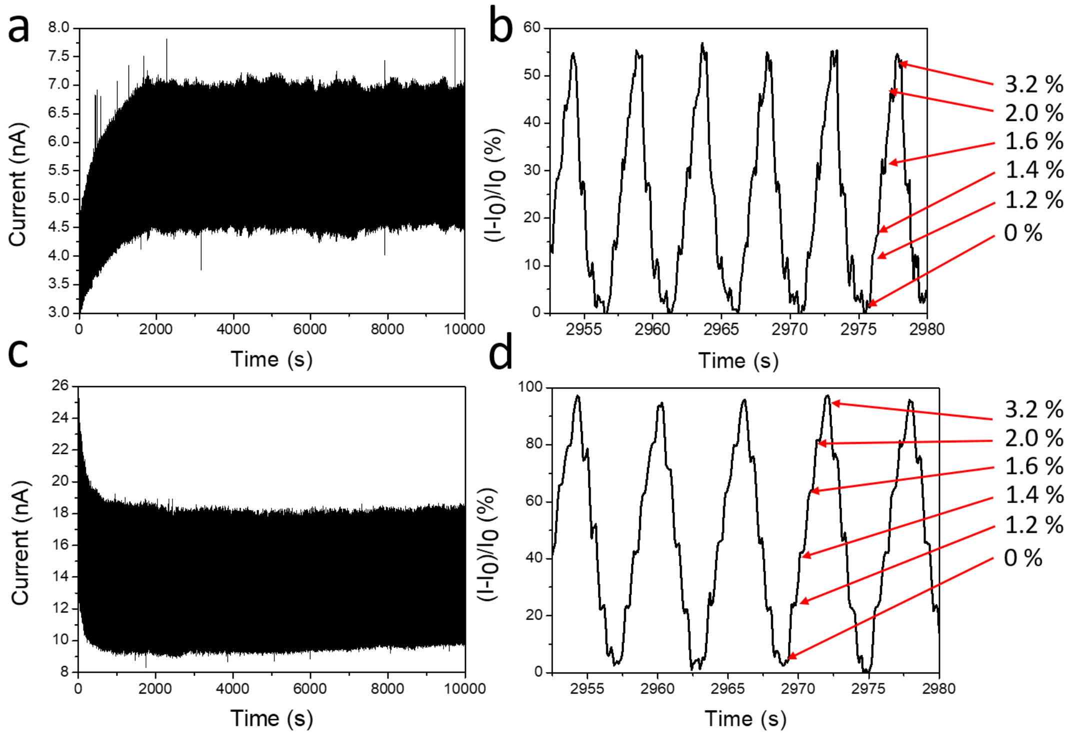

Supplementary Figure 19. Strain dependant current and resilience of a graphite/MoS_2/WS_2/graphite heterostructure on a PET substrate. a. Change in current with tensile strain at 5 V. **b.** A magnified section of **a.** highlighting the individual straining cycles. **c.** Change in current with compressive strain at 5 V. **d.** A magnified section of (iii) highlighting the individual strain cycles. The magnitude of applied strain at each point in the cycle is highlighted by the red arrow.

To determine the long-term durability of our strain sensors, we performed over 10^3 bending cycles for both tensile and compressive strain for a fixed bias voltage. As shown in Supplementary Figure 19, devices produced through the abrasive method are extremely robust, showing little-to-no change in current response to applied strain throughout the measured bending cycles.

Supplementary Figure 20, shows histograms for the change in current through the channel for both compressive and tensile strains of ~4%, as well as optical micrographs of an abraded heterostructure under tensile and compressive strain and in the relaxed state.

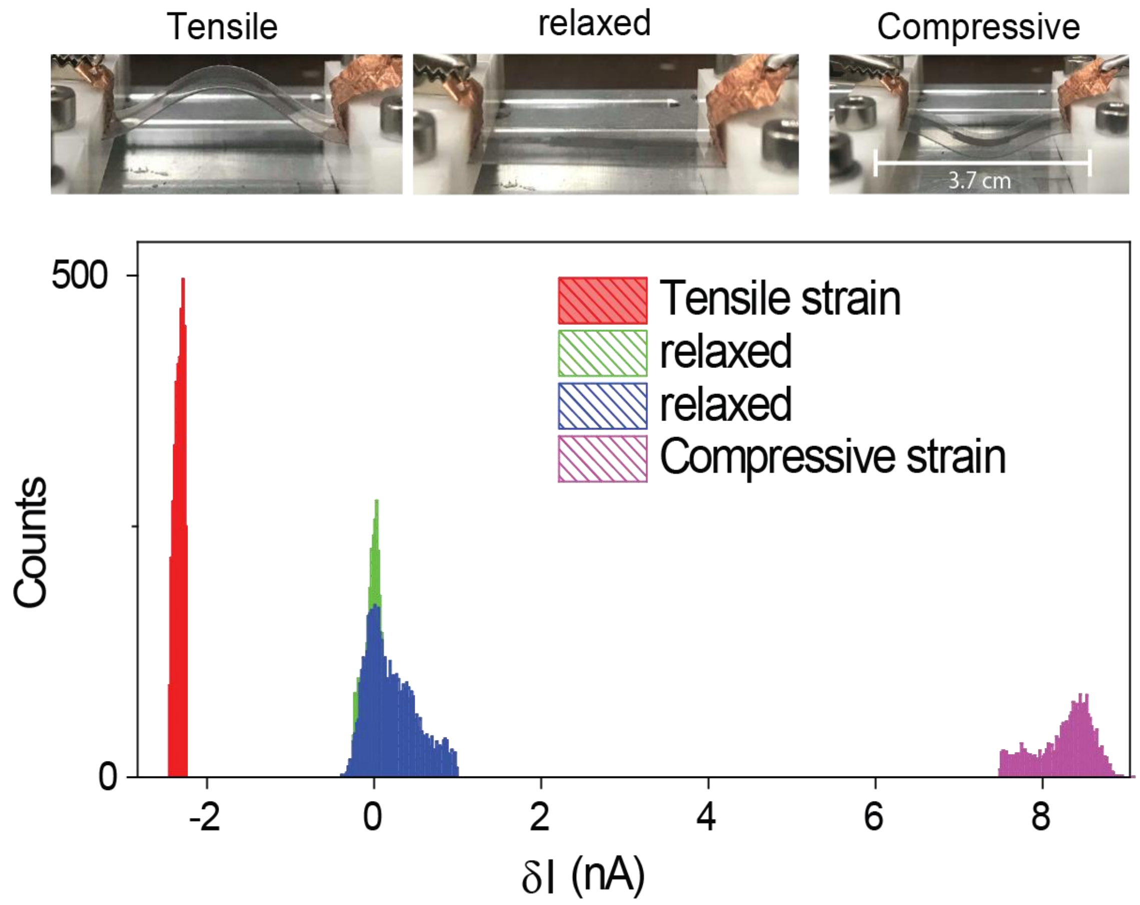

Supplementary Figure 20. Histogram of current response to strain of a graphite/MoS_2/WS_2/graphite heterostructure on a PET substrate. Compressive and tensile strain dependent changes in the current through a vertical graphite-MoS_2-WS2-graphite junction for over 10^3 bending cycles at a strain level of 4%.

Supplementary Note 11: Electrostatic gating of CVD graphene with abraded hBN dielectrics

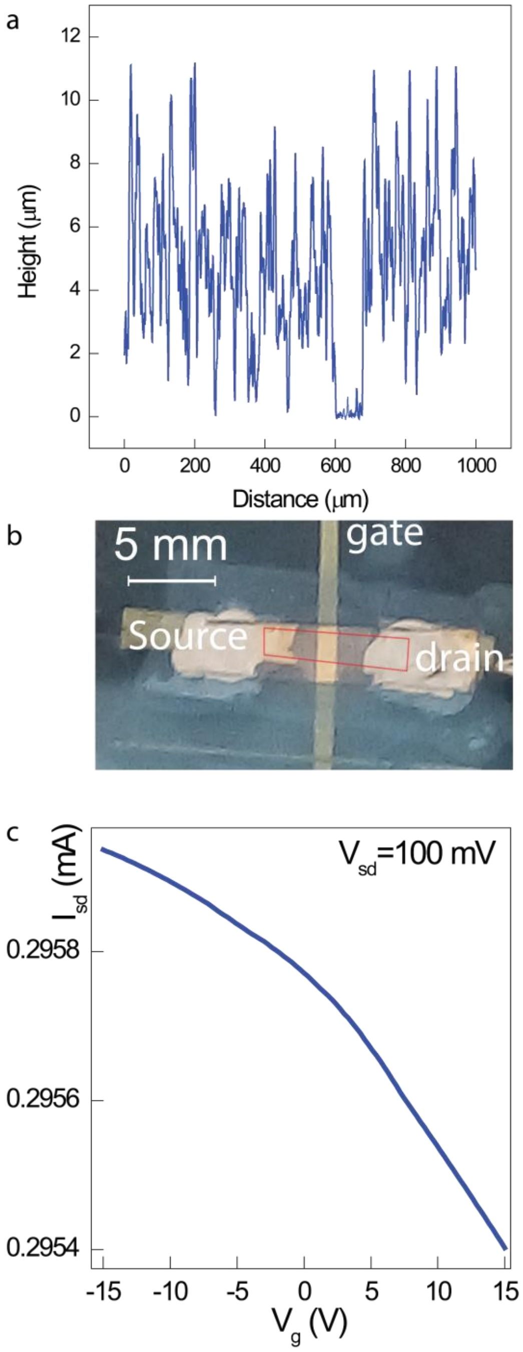

Supplementary Figure 21. a. Profilometry of the hBN film measured across a scratch in the film **b.** optical image of the hBN capacitor with the CVD graphene strip outlined in red. **c.** Electrostatic gating through abraded hBN films. Here V_g is applied to the Au gate electrode to modulate the source-drain current through the CVD graphene channel.

Supplementary Figure 21a shows the surface profile of a scratch near the region of the film used for the capacitance measurements which yields an average height of $5 \pm 2\ \mu m$. Fig. 21B shows a photo of the hBN capacitor device discussed in Fig. 1f, the red region highlights the CVD graphene strip which was transferred onto the hBN dielectric.

Supplementary Figure 21c, shows a weak gate dependence of the resistance of CVD graphene channel material vs applied gate voltage for the device shown in Fig. 2c of the main text. The weak dependence is likely due to the large variance of film thickness and large thickness of the hBN dielectric barrier.

Supplementary Note 12: Controlled abrasion of van der Waals powders via a CNC milling machine

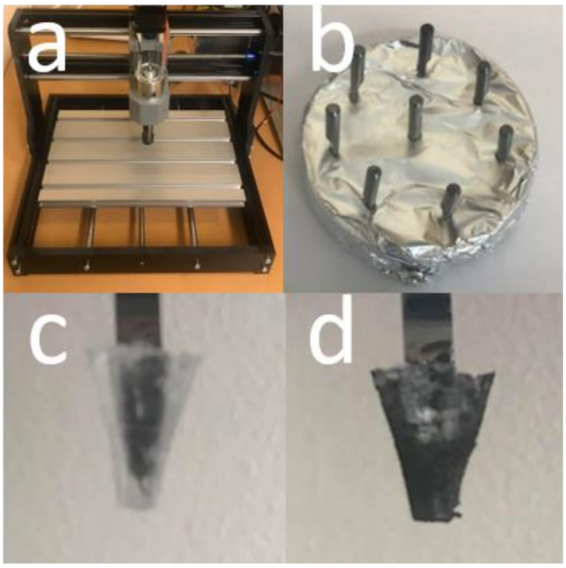

Supplementary Figure 22. Images showing equipment used to controllably abrade van der Waals powders. a. A CNC 3018 Pro milling machine used to abrade the films. **b.** Method used to produce a PDMS-capped drill bit. **c,d**. PDMS-capped drill bit before **c.** and after **d.** covering with graphite powder.

Supplementary Figure 22a, shows a CNC 3018 Pro milling machine which we used to investigate the electronic properties of abraded graphitic films produced in a more controlled and quantified manner than by hand. PDMS was created by mixing a 10:1 ratio of silicon elastomer base to curing agent, respectively. The mixture is left for 1 hour to allow for trapped air bubbles to escape. It was then covered in several layers of Al foil through which the drill bits are pressed. The tension in the Al foil serves to keep the drill bits upright and suspended in the PDMS mixture during the curing process. The PDMS is then baked at 80 °C for 3 hours until cured. Afterwards, a scalpel was used to remove the drill bits and to shape the PDMS cap

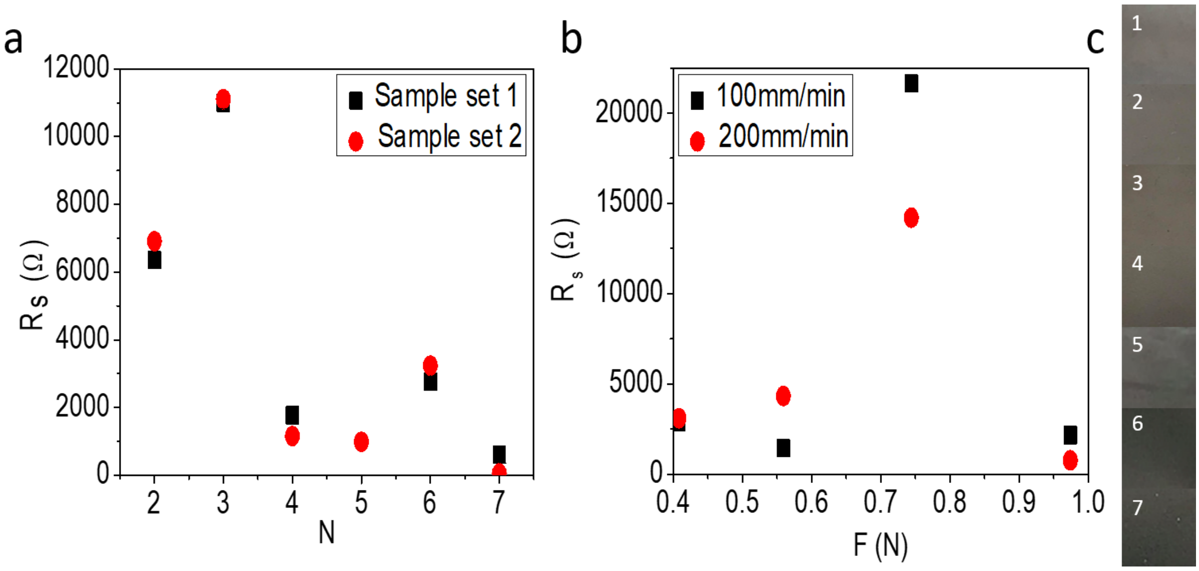

Supplementary Figure 23. Electrical characterisation of films abraded using a CNC milling machine. a. Sheet resistance, R_s, as a function of the number of passes, N, used to abrade a set of graphite films. **b.** Sheet resistance as a function of force applied by the PDMS-capped drill bit to the PET substrate and the feed rate (in mm/min). **c.** Image showing the colour of the abraded graphite as the thickness increases (indicated by the number of passes, 1-7).

(typically cut to be ~ 2mm thick on all sides). The PDMS cap is then covered in graphite (simply lightly pressing into graphite powder is enough) and used to write the pattern of choice.

The CNC miller was used to abrade graphite onto a 0.025 mm thick PET substrate following a pre-programmed pattern. The drill was set to 100 rpm during the production of all devices. Higher revolution speeds (up to the machine maximum of 1000 rpm) do not cause the PDMS cap to become detached from the drill bit. Furthermore, production and characterisation of devices were conducted in ambient conditions. Supplementary Figure 23a illustrates the abraded sheet resistance as a function of the "number of passes", N. This refers to the number of times that each abraded film was patterned in order to increase its thickness. As expected, the sheet resistance generally decreases as the thickness of the abraded graphite increases, to as low as 50 Ω at 7 passes. However, it should be noted that a single pass alone is not enough to abrade a continuous graphitic film and thus no conduction is seen in these samples. For both sample sets the sheet resistance spikes at 3 passes. This may be due to damage of the initial abraded film upon the second pass, which is then repaired as more graphite is added on subsequent passes. Supplementary Figure 23b illustrates sheet resistance as a function of force applied between the PDMS-capped drill bit and the PET substrate, F, for two different feed rates (100mm/min and 200mm/min). For each feed rates the sheet resistance spikes for an intermediate applied force of ~ 0.75 N.

Application of forces larger than ~ 1 N consistently caused the underlying PET substrate to tear. This was particularly prevalent at feed rates > 300 mm(min^{-1}) . This problem could be overcome by using a thicker PET substrate, although this may compromise its flexibility.

Supplementary Note 13: Stability of the material on the substrate

To determine how well the nanocrystals, adhere to the substrate, we submerged a graphitic film formed through 7 CNC write passes into tap water and left it submerged for 10 minutes. We compared the I-V_b characteristics before and after submersion in tap water. We find that the sheet resistance increases from 46 to 81 Ohms. This indicates that some material is removed due to the dipping in water, however we do not see a significant (order of magnitude) increase, indicating that most of the film remains intact as shown in the figure below.

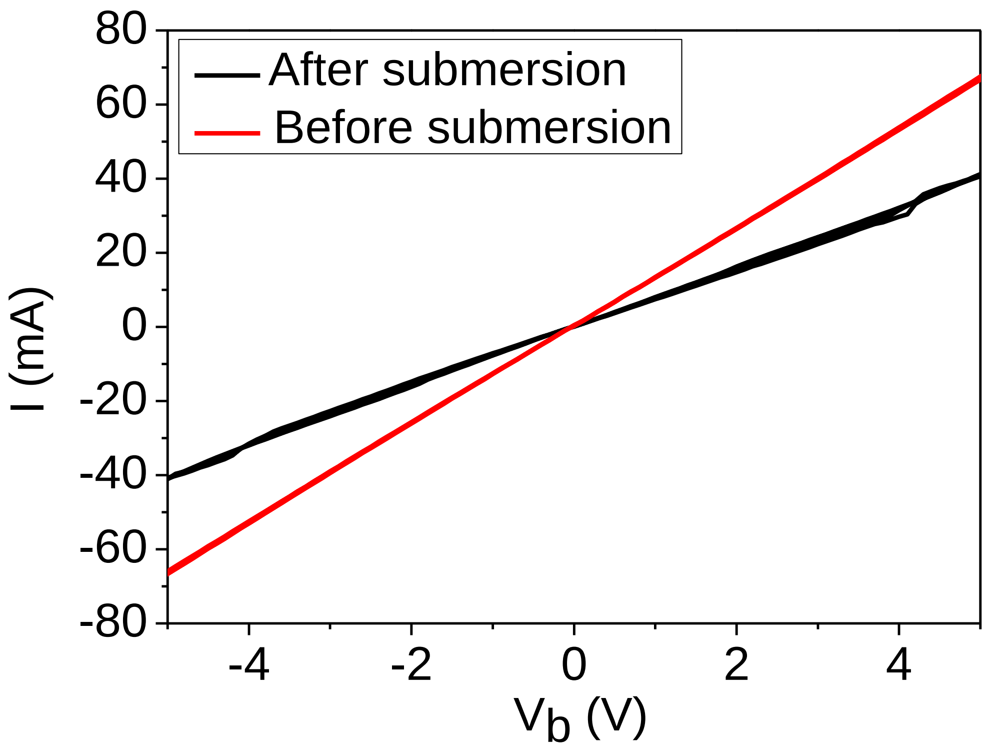

Supplementary Figure 24. Electrical characterisation of an abraded graphite film before and after submersion in tap water for 10 minutes.

Supplementary References.

1. Lotya M, Rakovich A, Donegan JF, Coleman JN. Measuring the lateral size of liquid-exfoliated nanosheets with dynamic light scattering. *Nanotechnology* **24**, (2013).

2. Bromley RA, Yoffe AD, Murray RB. Band Structures of Some Transition-Metal Dichalcogenides .3. Group Vi a - Trigonal Prism Materials. *J Phys Part C Solid* **5**, 759-& (1972).

3. Coehoorn R, Haas C, Degroot RA. Electronic-Structure of Mose2, Mos2, and Wse2 .2. The Nature of the Optical Band-Gaps. *Phys Rev B* **35**, 6203-6206 (1987).

4. Su ZX, Zhou WZ, Zhang Y. New insight into the soot nanoparticles in a candle flame. *Chem Commun* **47**, 4700-4702 (2011).

5. Niu Y, Frisenda R, Flores E, Ares JR, Jiao W, Perez de Lara D, Sanchez C, W. R, Ferrer IJ, Castellanos-Gomez A. Polarization-Sensitive and Broadband Photodetection Based on a Mixed-Dimensionality TiS3/Si p-n Junction. *Advanced Optical Materials* **6**, 1800351-1800351 (2018).

6. Withers F, Yang H, Britnell L, Rooney AP, Lewis E, Felten A, Woods CR, Romaguera VS, Georgiou T, Eckmann A, Kim YJ, Yeates SG, Haigh SJ, Geim AK, Novoselov KS, Casiraghi C. Heterostructures Produced from Nanosheet-Based Inks. *Nano Lett* **14**, 3987-3992 (2014).

7. Britnell L, Ribeiro RM, Eckmann A, Jalil R, Belle BD, Mishchenko A, Kim YJ, Gorbachev RV, Georgiou T, Morozov SV, Grigorenko AN, Geim AK, Casiraghi C, Castro Neto AH, Novoselov KS. Strong Light-Matter Interactions in Heterostructures of Atomically Thin Films. *Science* **340**, 1311-1314 (2013).

8. Gorbachev RV, Riaz I, Nair RR, Jalil R, Britnell L, Belle BD, Hill EW, Novoselov KS, Watanabe K, Taniguchi T, Geim AK, Blake P. Hunting for Monolayer Boron Nitride: Optical and Raman Signatures. *Small* **7**, 465-468 (2011).

9. Ferrari AC. Raman spectroscopy of graphene and graphite: Disorder, electron-phonon coupling, doping and nonadiabatic effects. *Solid State Commun* **143**, 47-57 (2007).

10. Ferrari AC, Meyer JC, Scardaci V, Casiraghi C, Lazzeri M, Mauri F, Piscanec S, Jiang D, Novoselov KS, Roth S, Geim AK. Raman spectrum of graphene and graphene layers. *Phys Rev Lett* **97**, (2006).

11. Zhang X, Tan QH, Wu JB, Shi W, Tan PH. Review on the Raman spectroscopy of different types of layered materials. *Nanoscale* **8**, 6435-6450 (2016).

12. Necas D, Klapetek P. Gwyddion: an open-source software for SPM data analysis. *Cent Eur J Phys* **10**, 181-188 (2012).

13. Khrapach I, Withers F, Bointon TH, Polyushkin DK, Barnes WL, Russo S, Craciun MF. Novel Highly Conductive and Transparent Graphene-Based Conductors. *Adv Mater* **24**, 2844-2849 (2012).

14. Shin DW, Barnes MD, Walsh K, Dimov D, Tian P, Neves AIS, Wright CD, Yu SM, Yoo JB, Russo S, Craciun MF. A New Facile Route to Flexible and Semi-Transparent Electrodes Based on Water Exfoliated Graphene and their Single-Electrode Triboelectric Nanogenerator. *Adv Mater* **30**, (2018).

15. Ovchinnikov D, Allain A, Huang Y-S, Dumcenco D, Kis A. Electrical Transport Properties of Single-Layer WS2. *Acs Nano* **8**, 8174-8181 (2014).

16. Chen T, Sheng Y, Yingqui Z, Chang R-J, Wang X, Huang H, Zhang Q, Hou L, Warner JH. High Photoresponsivity in Ultrathin 2D Lateral Graphene:WS2:Graphene Photodetectors Using Direct CVD Growth. *ACS Appl Mater Interfaces* **11**, 6421-6430 (2019).

17. Kam K-K. Electrical properties of WSe2, WS2, MoSe2, MoS2, and their use as photoanodes in a semiconductor liquid junction solar cell. *The dissertation thesis, Iowa State University,* (1982).

18. Finn DJ, Lotya M, Cunningham G, Smith RJ, McCloskey D, Donegan JF, Coleman JN. Inkjet deposition of liquid-exfoliated graphene and MoS2 nanosheets for printed device applications. *J Mater Chem C* **2**, 925-932 (2014).

19. Cunningham G, Hanlon D, McEvoy N, Duesberg GS, Coleman JN. Large variations in both dark- and photoconductivity in nanosheet networks as nanomaterial is varied from MoS2 to WTe2. *Nanoscale* **7**, 198-208 (2015).

20. McManus D, Dal Santo A, Selvasundaram PB, Krupke R, LiBassi A, Casiraghi C. Photocurrent study of all-printed photodetectors on paper made of different transition metal dichalcogenide nanosheets. *Flex Print Electron* **3**, (2018).

21. Georgiou T, Jalil R, Belle BD, Britnell L, Gorbachev RV, Morozov SV, Kim YJ, Gholinia A, Haigh SJ, Makarovsky O, Eaves L, Ponomarenko LA, Geim AK, Novoselov KS, Mishchenko A. Vertical field-effect transistor based on graphene-WS2 heterostructures for flexible and transparent electronics. *Nat Nanotechnol* **8**, 100-103 (2013).

22. McManus D, Vranic S, Withers F, Sanchez-Romaguera V, Macucci M, Yang HF, Sorrentino R, Parvez K, Son SK, Iannaccone G, Kostarelos K, Fiori G, Casiraghi C. Water-based and biocompatible 2D crystal inks for all-inkjet-printed heterostructures. *Nat Nanotechnol* **12**, 343-350 (2017).
